# The sources of high airborne radioactivity in cryoconite holes from the Caucasus (Georgia)

**DOI:** 10.1038/s41598-018-29076-4

**Published:** 2018-07-17

**Authors:** Edyta Łokas, Krzysztof Zawierucha, Anna Cwanek, Katarzyna Szufa, Paweł Gaca, Jerzy W. Mietelski, Ewa Tomankiewicz

**Affiliations:** 1 Department of Nuclear Physical Chemistry, Institute of Nuclear Physics Polish Academy of Sciences, Kraków, Radzikowskiego 152 31-342 Poland; 2 Department of Animal Taxonomy and Ecology, Adam Mickiewicz University, Poznań, Poland; 3 GAU-Radioanalytical Laboratories, Ocean and Earth Science, University of Southampton, National Oceanography Centre, European Way, Southampton United Kingdom

## Abstract

Cryoconite granules are mixtures of mineral particles, organic substances and organisms on the surface of glaciers where they decrease the ice albedo and are responsible for formation of water-filled holes. The contaminants are effectively trapped in the cryoconite granules and stay there for many years. This study evaluates the contamination level of artificial and natural radionuclides in cryoconite holes from Adishi glacier (Georgia) and identifies the sources of contamination based on activity or mass ratios among artificial radionuclides. Results revealed high activity concentrations of fallout radionuclides reaching 4900 Bq/kg, 2.5 Bq/kg, 107 Bq/kg and 68 Bq/kg for ^137^Cs, ^238^Pu, ^239+240^Pu and ^241^Am, respectively. The main source of Pu is global fallout, but the low ^240^Pu/^239^Pu atomic ratios also indicated local tropospheric source of ^239^Pu, probably from the Kapustin Yar nuclear test site. Also, high activity ratios of ^241^Am/^239+240^Pu could originate from Kapustin Yar. The natural radionuclides originate from the surrounding rocks and were measured to control the environmental processes. ^210^Pb in cryoconite granules comes predominantly from the atmospheric deposition, and its activity concentrations reach high values up to 12000 Bq/kg.

## Introduction

Glaciers and ice sheets cover ca. 10% of the surface of the land and constitute an extreme biome where 70% of global freshwater reservoirs are stored^1–4^. Because of climate change and the related cryosphere decline, glaciers constitute potentially important natural hazards as main contributors to sea level rise as well as when they collapse from mountain slopes into inhabited areas^1,2,5,6^. Moreover, glaciers are economically important as sources of the hydroelectric power production for domestic use and the only sources of domestic water in different parts of the world^7^. Also, they may be the source of potential pathogens, black carbon, persistent organic pollutants and even an antibiotic-resistant bacteria^8–13^. Recent studies show that glaciers may be the source of other threats, e.g. the release into the environment of anthropogenic and natural radionuclides. Indeed, glaciers were recognized as repositories for such substances. Because of ice melting, radionuclides stored in glaciers may be transported to downstream ecosystems and be accumulated in biota, with further consequences along the trophic chain^11,14^. The main sources of anthropogenic radionuclides in the Northern hemisphere are (i) nuclear weapon tests and atmospheric explosions in Novaya Zemlya, Semipalatinsk, and Nevada; (ii) nuclear accidents (Kyshtym-1957, Lake Karchay-1968; Tomsk-1993; Chernobyl-1986 or Fukushima-2011); and (iii) disintegrations of satellites (SNAP9A-1964, Cosmos 958–1978). In the case of the Caucasian or Black Sea areas, a small and less investigated former Soviet local test site at Kapustin Yar where five small-yield (10–40 kt) atmospheric tests were performed in 1957–61^15^ should also be considered.

**Figure 1:**
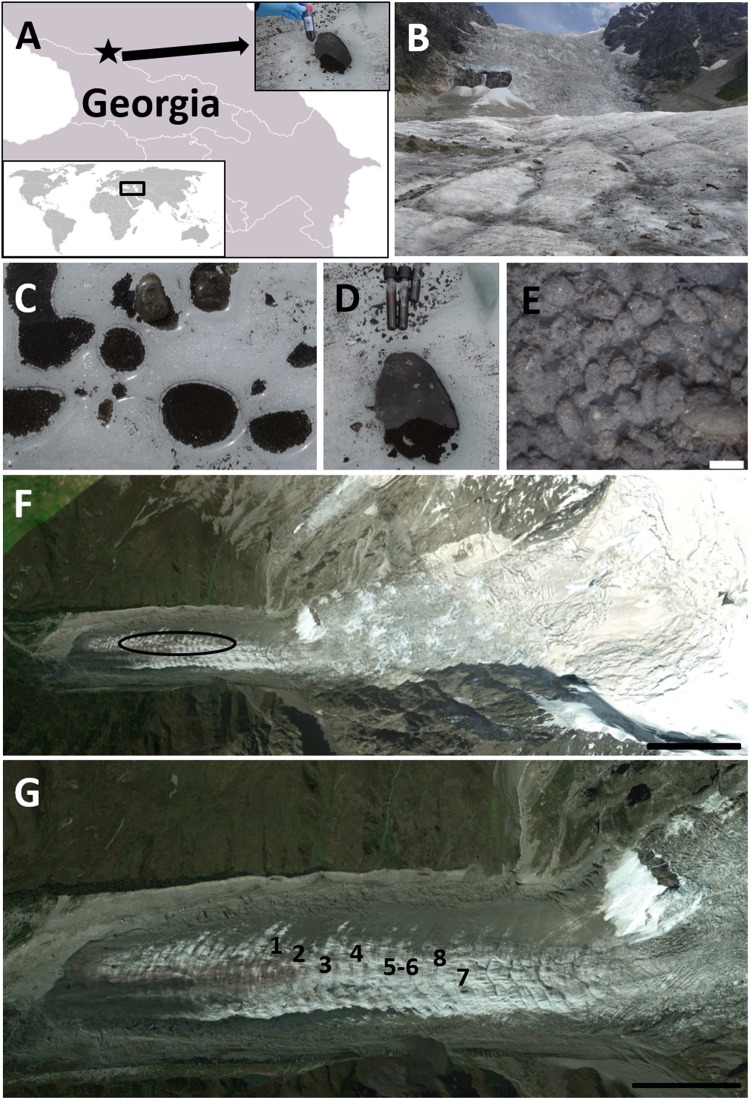
Study area. Map of the Georgia: star indicates sampling area, inserted picture shows the cryoconite hole on Adishi Glacier with a map of the World: frame indicate Caucasus region and Georgia(**A**) (Permission for using figures is granted to copy, distribute and/or modify this document under the terms of the GNU Free Documentation License (https://commons.wikimedia.org/wiki/Commons:GNU_Free_Documentation_License,_version_1.2). The links to original files are https://commons.wikimedia.org/wiki/File:BlankMap-Caucasus.png#filelinks (under licence https://creativecommons.org/licenses/by-sa/3.0/deed.en), https://commons.wikimedia.org/wiki/File:BlankMap-World-v2.png (under licence https://creativecommons.org/licenses/by-sa/3.0/deed.en) respectively); Adishi Glacier where the samples were collected (**B**); cryoconite holes from Adishi Glacier (2 **C**,**D**); cryoconites granules, scale bar 0.5 mm (2 **E**); view of Adishi Glacier (source: Google Earth; US Dept of State Geographer ©2018 Google, Image Landsat/Copernicus, Image ©2018 DigitalGlobe), scale bar 0.5 km (**F**); view of Adishi Glacier tongue with marked sampling points (source: Google Earth), scale bar 0.25 km (**G**) (all black shapes and description were added to original pictures).

The highest concentrations of pollutants on glaciers are most likely stored in cryoconite granules and micro-fauna^11,13,14^. Cryoconite granules are aggregates of mineral and organic components which form biological consortia with archaea, algae, cyanobacteria, fungi and heterotrophic bacteria^4,16–18^. Cyanobacteria play a crucial role in the formation of cryoconite granules, and they produce extracellular polymeric substances whose adhesive properties enhance the accumulation of both dust and microorganisms^4,18,19^. The dark-colored humic substances, residues from bacterial decomposition and organic matter are some of the causes of the glacier surface darkening which leads to albedo reduction, influencing the formation of cryoconite holes and water-filled reservoirs on glaciers^16–18^. In addition, they usually form in ablation zones of glaciers. Also, due to the relatively high concentration of nutrients to the availability of liquid water, the ablation zones represent a hotspot for biodiversity in glacial environments^4,18,20,21^. Micro-fauna found in the cryoconite holes, such as rotifers (Rotifera) and tardigrades (Tardigrada), play the role of grazers and may accumulate pollutants as apex consumers^11,14^. Several papers have been published in recent years on the diversity of the organisms flourishing in cryoconite, on their potential negative impact as pathogens, on the interaction between ice and organisms, on the darkening of ice and on the connection with algal blooms^4,10,21–25^. Other aspects which were investigated related to the biotechnological and astrobiological potentials of cryoconite holes and their inhabitants^26–28^. Polar glaciers are intensively investigated in both Arctic and Antarctic; however, little attention has been given to the mountain glaciers^29,30^, especially in the Eurasian area. Those glaciers are projected to lose 80% of their volume by the end of 2100, and some of them are expected to disappear within decades at current climatic conditions^31^. The best example is the Caucasus glaciers, with mostly glaciological papers published so far^32–34^, and only one single paper studies the microorganisms and analyses the total microbial 16 S rRNA gene in cryoconite sediments, ice and gravel^30^. The analysis of radionuclide contaminations in the inland Caucasus glacier (Fig. 1) is essential because the position of Caucasus is near many of the most important sites where nuclear activities are not explored sufficiently. The artificial radionuclides that are studied in this article (^137^Cs, ^238,239,240^Pu, ^241^Am, ^90^Sr) were introduced into the environment in the second half of the 20th century. However, the estimates of their atmospheric deposition in Eastern Europe are fragmented and inaccurate. Therefore, there is a need for methodical and primary research on the contents of these radionuclides in cryoconite which can preserve information on their atmospheric deposition. Moreover, the Fukushima Power plant accident in 2011 has revived the interest of the researchers and the general public in the consequences of the releases and global spreading of radioactive contamination.

Besides the global fallout from nuclear explosions, which is the result of a worldwide, mainly stratospheric, transport of contaminants, the influence of a tropospheric transport occurring at much shorter distances may also be considered. Isotopic ratios characteristic for the nuclear test site can differ from those for the global fallout. Among the radioactive contaminants, the most dangerous are believed to be alpha emitters (^241^Am and Pu isotopes) due to their long physical half-life and high biological toxicity. ^241^Am originates mainly from the decay of ^241^Pu released into the environment, and its activity increases with time^35^. The main objective of this study is to determine the activity concentrations of anthropogenic (^137^Cs, ^238,239,240^Pu isotopes, ^241^Am, ^90^Sr) and natural radionuclides (^210^Pb, ^230,232^Th and ^234,238^U isotopes) and identify their sources on Adishi glacier in the Caucasus.

## Results and Discussion

**Figure 2:**
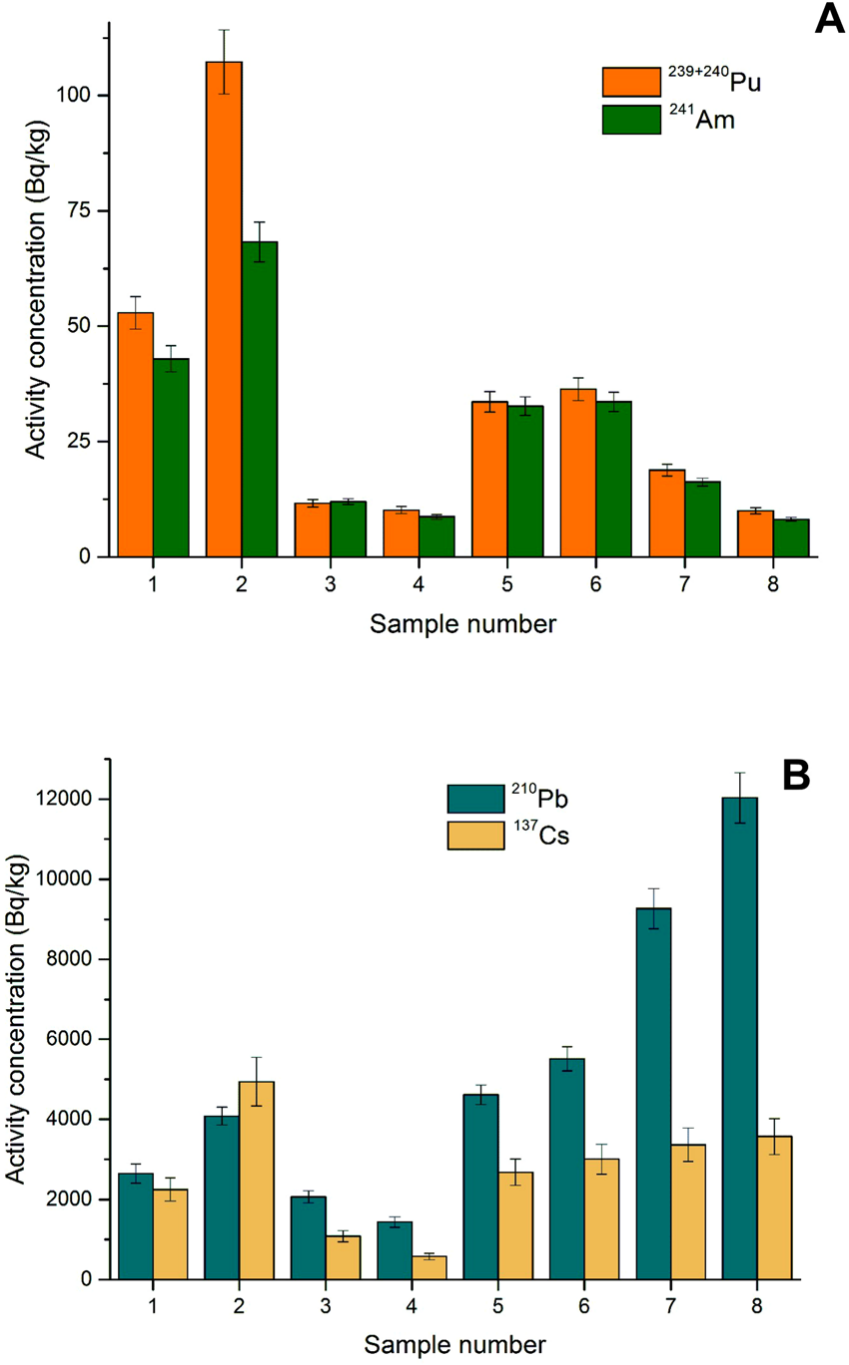
Activity concentrations of airborne radionuclides (^239+240^Pu, ^241^Am, ^90^Sr) (2**A**) and (^137^Cs and ^210^Pb) (2**B**) in cryoconite samples.

**Figure 3:**
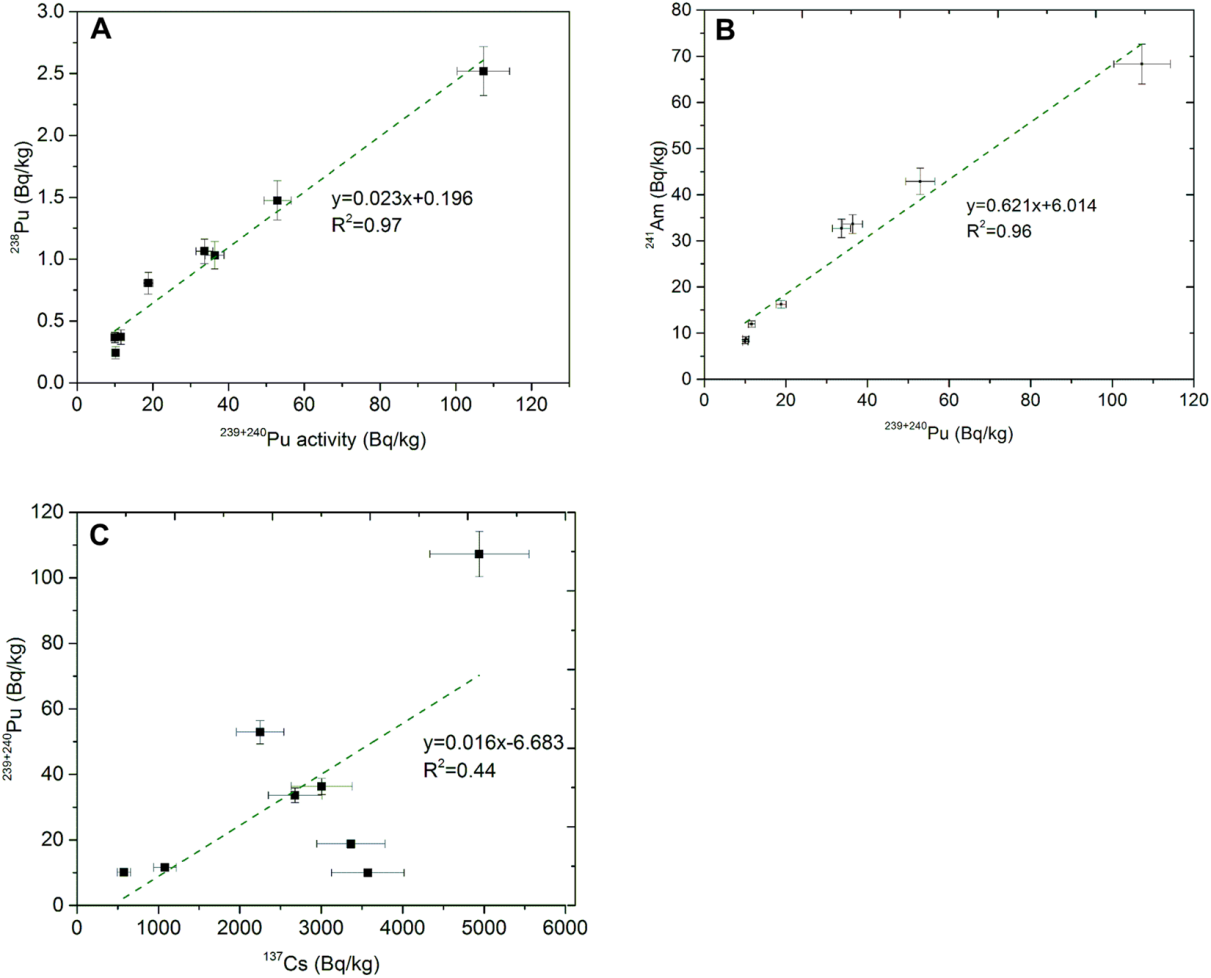
Regression plots for Pu isotopes (3 **A**) vs Am (3 **B**) and Cs (3 **C**) for all cryoconite samples.

**Figure 4:**
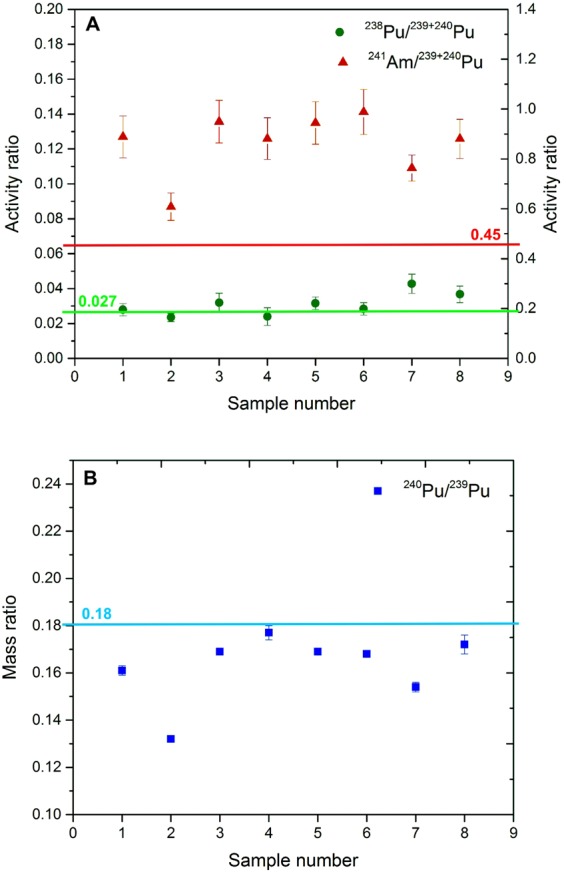
Activity ratios (4 **A**) and atomic ratios (4 **B**) for all cryoconite samples with reference lines.

**Figure 5:**
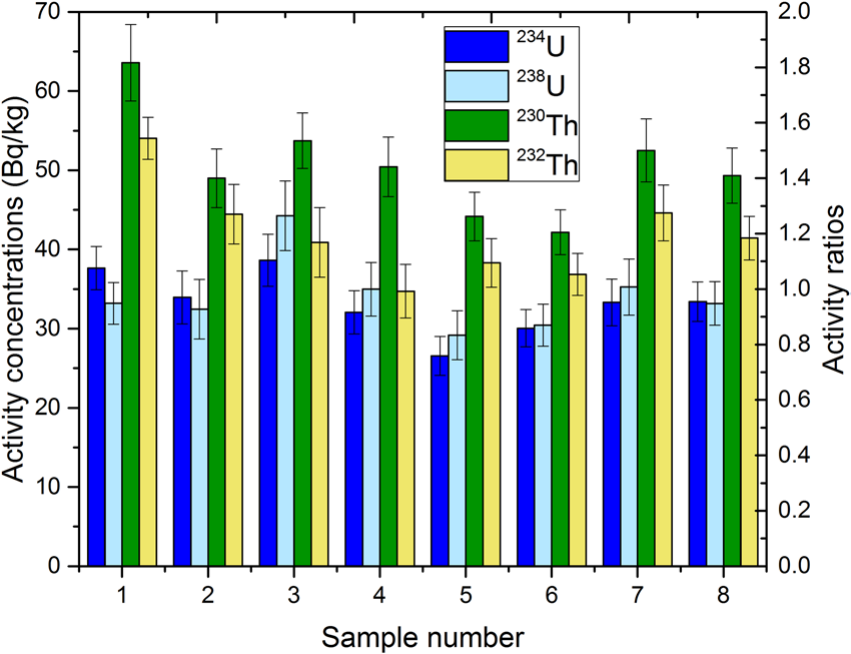
Activity concentrations of natural radionuclides (U and Th isotopes) in all cryoconite samples.

Data for all analysed radionuclides are presented in the Supplementary Material. Graphical results are presented in Figs 2A,B; 3A–C; 4A,B and 5.

### Artificial radionuclides and their activity ratios

The results of activity concentrations of anthropogenic radionuclides (^137^Cs, ^238^Pu, ^239+240^Pu, ^241^Am, ^90^Sr) for all the cryoconite samples are presented in Table 1 (Supplementary data) and Fig. 2. Activity concentrations range from: 580 ± 80 to 4940 ± 610 Bq kg^−1^ for ^137^Cs, 0.37 ± 0.04 to 2.52 ± 0.20 Bq kg^−1^ for ^238^Pu, 10.0 ± 0.7 to 107.3 ± 6.9 Bq kg^−1^ for ^239+240^Pu, 8.1 ± 0.4 to 68.3 ± 4.3 Bq kg^−1^ for ^241^Am and 23 ± 2 to 97 ± 8 Bq kg^−1^ for ^90^Sr (^137^Cs and ^90^Sr decay corrected to 15 August 2014, the sampling date). Data about artificial radionuclides in this region are scarce and poorly investigated, especially in case of ^241^Am and ^90^Sr, and only three papers^36–38^ report Pu and Am measurements in the Black Sea region. High values for ^137^Cs contaminations were observed in soils from West Georgia with the maximum value of 1279 Bq kg^−1 ^^39^, but Urushadze and Manakhov (2017)^36^ reported activity concentrations of ^137^Cs significantly lower (maximum value is 144 Bq kg^−1^).

The highest activity concentrations for all considered artificial radionuclides were observed in sample 2, except ^90^Sr, where the highest activity concentration was in sample 1. Such high activity concentrations of these radionuclides were reported only in cryoconite samples from other sites (Alpine glaciers, Svalbard glacier)^11,13,40,41^. The ability of cryoconite material to retain and concentrate the airborne radionuclides and metals could be related to metal binding properties of extracellular substances that are excreted by microorganisms (cyanobacteria) to immobilize metallic contaminants^42,43^. In cryoconite holes on Hans Glacier (SW Spitsbergen) along with radionuclides and heavy metals, high densities of micro-animals were detected^11^. In turn, on the edge of Greenland Ice Sheet, low radionuclides content seems to be the result of strong flushing, lack of animals and erosion of granules^14^. On Adishi Glacier, micro-fauna were detected in the holes^30^, and they, along with the presence of granules (mostly cyanobacteria), may store contaminants. Differences in activity concentrations of airborne radionuclides (^238,239,240^Pu, ^241^Am, ^90^Sr and ^210^Pb) found among the samples seems to reflect the location of sampling sites and the amount of organic matter. Samples 1 and 2 contain the highest value of organic matter (11 and 16%) and ^137^Cs, Pu isotopes and ^241^Am in these samples were the highest. These sampling points (1 and 2) were located closer to the moraine (Fig. 1), and both sampling points were surrounded by debris covered ice surfaces. Sample 1 resembles typical oval cryoconite hole but sampling point 2 was characterized by the presence of gravel at the cryoconite hole bottom. The highest activity concentrations of ^210^Pb are detected in samples 5, 6, 7 and 8. These samples were collected from typical cryoconite holes with dark and dense cryoconite material on the bottom (Fig. 1C,D). The role of the cryoconite systems in sediment transfers downstream the glacier is not fully understood. Regardless of the lifespan of individual cryoconite holes, their collapse does not imply removal of cryoconite from glacier surface as the dispersed cryoconite granules initiate formation of new holes^16^. The cryoconite material could be retained in the porous weathering crust that develops on the surfaces of non-temperate glaciers^44,45^. Migration of supraglacial streams was proposed as the only effective mechanism of cryoconite evacuation from glacier surfaces^4^. Once washed down by a stream, the cryoconite granules enter the supraglacial or subglacial drainage system and, even if deposited in the glacier forefront, they become diluted by the prevailing sediments with low artificial radionuclide contents. Occurrences of the cryoconite-derived material with high radionuclide contents in the glacier forefronts indicate that the cryoconite granules can be retained on the glacier surface or in a deeper ice layer and deposited at the glacier terminus after the ice melts out. Samples 5–8 were collected between ice waterfall and the edge of ice tongue where the flatty surface dominated and higher deposition of ^210^Pb was observed. In these samples, Pu isotopes and ^241^Am show much lower activity concentrations. The cryoconite granules could disintegrate on the surface of the glacier; therefore, the activities of Pu isotopes, ^241^Am and ^90^Sr are diluted, while activity concentrations of ^210^Pb are much higher due to the long exposure of the material to the atmosphere. Levels of ^210^Pb and artificial radionuclides contents in cryoconite granules reflect the temporal patterns of their exposure to atmospheric deposition. Because of the constant delivery of ^210^Pb from the atmosphere concentration of this radionuclide in cryoconite material should be proportional to the exposure time, while high concentrations of the artificial radionuclides indicate significant contribution of material that was exposed to the stratospheric or tropospheric fallout. Cryoconite granules have infrequent, often patchy distribution which supports long residence on the ice surface of some glaciers^4,44^. Prolonged exposure of cryoconite to the atmospheric dust may then lead to the build-up of the radionuclide contents to high levels. Using ice cores samples, Segawa *et al*.^46^ proved that cyanobacteria found on glaciers remained unchanged in last 12,500 years. Moreover, Segawa *et al*. (2018) showed that cyanobacterial population sizes increased during the Holocene^46^, corroborating with our hypothesis regarding the prolonged exposure and increasing accumulation of contaminants.

The artificial radionuclides could originate from various sources mentioned previously; however, the fresh and relatively intense fallout occurs in isolated events. Between them (Pu isotopes, ^241^Am, ^137^Cs and ^90^Sr), mostly traces are deposited from resuspension. In contrast, airborne natural radionuclides, such as ^210^Pb, are deposited at a more constant rate. The diminishing content of ^210^Pb suggests that the majority of material in samples 1–4 was already removed with melt waters, or these materials can be covered by ice and had no contact with the atmosphere. Samples 1 and 2 had the highest activity concentrations of artificial radionuclides which originate from the original deposition. After this event, these materials can be redeposited in places where the deposition of ^210^Pb was limited. In contrast, sampling points 1 and 2 were localised close to the moraine, and this glacial area is usually covered with debris originating from the moraine. Thus, artificial radionuclides may come from moraine material that is removed from the glacier tongue during geomorphological processes and may be stored in water reservoirs in the debris-covered area of the glacier.

The activity ratios of ^238^Pu/^239+240^Pu, ^239+240^Pu/^137^Cs and ^241^Am/^239+240^Pu, as well as atomic ratios of ^240^Pu/^239^Pu, were calculated to distinguish the sources of these radionuclides in cryoconite samples.

The correlation factors between plutonium isotopes and ^137^Cs and ^241^Am concentrations (activity ratios of ^238^Pu/^239+240^Pu, ^239+240^Pu/^137^Cs and ^241^Am/^239+240^Pu) in cryoconite samples are presented in Table 2 (Supplementary data) and Fig. 3A–C and Fig. 4A. The ^238^Pu/^239+240^Pu activity ratios varied between 0.023 ± 0.002 (sample 2) and 0.043 ± 0.005 (sample 7). There are significant differences in activity concentrations between the lowest and the highest values of ^239+240^Pu; therefore, linear regression is an appropriate method to evaluate the correlation between investigated isotopes. The ^238^Pu and ^239+240^Pu activity concentrations are plotted versus each other (Fig. 3A). The slope of the best fit line (R^2^ = 0.97) equals 0.023 ± 0.002.

There are two main sources of plutonium in the Northern Hemisphere: global fallout with a ^238^Pu/^239+240^Pu activity ratio of 0.027 (calculated for 2014^47^) and spent fuel sources (e.g. waste from nuclear fuel reprocessing plants or Chernobyl accident). There is no clear and reasonable way to explain how the waste from reprocessing plants could reach mountain glaciers and influence the composition of cryoconite. The only probable source other than the global fallout from atmospheric tests is the Chernobyl accident, and it is characterized by the ^238^Pu/^239+240^Pu activity ratio of 0.45 in 2014^48^ or 0.33 (calculated from data given by Kudryashov *et al*.^47^). The activity ratio of ^238^Pu/^239+240^Pu was used to evaluate the percentage of Chernobyl-derived ^239+240^Pu activity. The model assumes that the ^238^Pu/^239+240^Pu activity ratios for global fallout and Chernobyl plutonium are about 0.027 and 0.45 (0.33), respectively. Calculations were performed for each of the studied samples, and the fraction of global fallout varied from 96 ± 1% to 100 ± 1%. Differences resulting from using different ^238^Pu/^239+240^Pu activity ratio for Chernobyl contribution (0.45) are approx. 1%, demonstrating that vast majority of the total Pu found in the samples comes from the nuclear weapons explosions (stratospheric global fallout and perhaps local tropospheric fallout) with only negligible fraction which may be attributed to the Chernobyl nuclear accident.

The analysis of atomic ratios of ^240^Pu/^239^Pu provides important information which allows more precise identification of the origin of Pu isotopes in environmental samples. The average atomic ratios of ^240^Pu/^239^Pu from global fallout are about 0.180 ± 0.007, and they depend on weapon design or explosion yield^49,50^. In addition, ^240^Pu/^239^Pu atomic ratios found in cryoconite samples varied from 0.132 ± 0.001 to 0.177 ± 0.003 (Fig. 4B) with an average of 0.163 ± 0.014 (1σ), indicating that there may be two different sources of the plutonium derived from weapon explosions in the analysed cryoconite samples. The measured ratios could be the result of mixing of both the stratospheric and tropospheric fallouts. The presence of the tropospheric fallout can be assumed by the presence of samples with ^240^Pu/^239^Pu atomic ratios close to 0.13, explaining the overall lower than 0.18 values for this ratio, especially those ranging from 0.132 to 0.154. The low ^240^Pu/^239^Pu atomic ratios potentially point to the explosion of a low-yield nuclear devices^51^, where the production of ^240^Pu is limited due to a relatively low neutron flux. The Soviet Union conducted a few low-yield, high-altitude nuclear tests in the Kapustin Yar nuclear test site, which is located relatively close to the Caucasus. These nuclear explosions, apparently tested as potential anti-aircraft warheads, may be responsible for this slightly unusual fallout. In addition, the presence of plutonium of such low ^240^Pu/^239^Pu atomic ratio could also potentially partially mask (compensate) the higher ratio Pu of the Chernobyl origin; however, the low ^238^Pu/^239+240^Pu activity ratio shows that the Chernobyl Pu in this case is insignificant. The ^239+240^Pu/^137^Cs activity ratios in the cryoconite samples range between 0.003 ± 0.001 to 0.024 ± 0.003. The slope of the best fit line (R^2^ = 0.44) in the ^239+240^Pu–^137^Cs correlation plot equals 0.016 ± 0.007 (Fig. 3C). The ratios are much lower than the decay-corrected value of 0.031 in the global fallout expected for the year 2014^52^, but the possibility that some of the ^137^Cs activity coming from the Chernobyl accident could not be excluded. Because of the long distance from Chernobyl where the ratio between ^137^Cs and ^239+240^Pu was high, the measurable amount of ^137^Cs in the analysed samples did not increase Pu contamination enough to influence the observed ^239+240^Pu/^137^Cs activity ratio. This ratio observed near the Chernobyl zone was estimated as 0.0088^53^. However, over the distance between Chernobyl and Central Europe, the ratio dropped to the level of 10^−5 ^^54^. The radionuclide ratio in the Chernobyl clouds depended on the release history, physical-chemical nature of the released matter and the atmospheric transport conditions, which mainly are the difference in aerosols diameters transporting Pu and Cs^55^. ^239+240^Pu/^137^Cs activity ratio for analysed samples are also lower than in cryoconite samples from Svalbard^11^, where the ratio varied between 0.011 to 0.030. The ^241^Am/^239+240^Pu activity ratios range between 0.64 ± 0.06 and 1.03 ± 0.09 (Fig. 4A). The slope of the best fit line (R^2^ = 0.96) in the ^241^Am - ^239+240^Pu correlation plot equals 0.62 ± 0.05 (Fig. 3B). Such high activity ratios (about 1) was found in the North-western Black Sea^37^, but in this region, ^238^Pu/^239+240^Pu activity ratios and ^240^Pu/^239^Pu atom ratios also exceeded the stratospheric fallout ratio because of the riverine transport mechanism. The transfer of the airborne contaminants in cryoconite can occur only by atmospheric deposition. The ^241^Am/^239+240^Pu activity ratio for global fallout is 0.45, but for the Chernobyl accident, the ratio is 2.2 (calculated for 2014 from data given by Kudryashov *et al*.^47^). No data is available on the Am/Pu ratio for Kapustin Yar explosions, but there is no clear reason why it should be higher than for global fallout. Since the presence of plutonium of Chernobyl origin was negligible in the samples, this should be similar for americium.

The mean activity ratios of ^90^Sr/^137^Cs and ^90^Sr/^239+240^Pu were 0.023 ± 0.014 and 2.06 ± 0.96, respectively. These ratios are much lower than the global fallout signatures of 0.641 for ^90^Sr/^137^Cs and 36 for ^90^Sr/^239+240^Pu. Strontium can be quickly removed from cryoconite samples because of its high solubility in water. Apparently, radiocesium and plutonium isotopes have much higher concentrations in cryoconite material then strontium due to the presence of both organic matter and traces of fine mineral fraction. The obtained results are also lower than in cryoconite samples from Arctic glacier^11^.

### Natural radionuclides

Data on natural radioisotopes (^210^Pb, ^234,238^U, ^230,232^Th and activity ratio of ^234^U/^238^U) for the cryoconite samples are presented in Table 3 (Supplementary material) and Fig. 5. Activity concentrations range from 1400 ± 100 to 12000 ± 600 Bq kg^−1^ for total ^210^Pb, 27 ± 2 to 39 ± 3 Bq kg^−1^ for ^234^U, 293 to 44 ± 4 Bq kg^−1^ for ^238^U, 49 ± 4 to 64 ± 5 Bq kg^−1^ for ^230^Th and 35 ± 3 to 54 ± 5 Bq kg^−1^ for ^232^Th. Activity concentrations of natural radionuclides (U and Th isotopes) show little variability between samples and do not differ from values reported for soils globally^56^. Activity concentrations of these lithogenic radionuclides are related to their contents in the source minerals^57^ with the exception of ^210^Pb which originates from two sources: *in situ* production from ^226^Ra decay products (supported) and from atmospheric deposition (unsupported). The unsupported ^210^Pb originates in the atmosphere from radioactive decay of the gaseous ^222^Rn, attaches itself to aerosol particles and is finally deposited with both wet and dry precipitation in a similar way to the artificial fallout radionuclides. The highest activity concentration for ^210^Pb is in sample 8 (Fig. 2B). Most of the airborne radioactivity of ^210^Pb is attached to aerosol particles, and that is why the activities accumulated in cryoconite granules, mosses and lichens, which could be exposed to atmospheric wet/dry deposition for a long time, may reach very high values^58^. Our previous paper^41^ showed similar results for the concentration of ^210^Pb (from 4000 to 9500 Bq kg^−1^) in cryoconite granules from the Werenskiold glacier (Svalbard, Arctic), while on the neighbouring Hans glacier^11^, they reach up to around 4600 Bq kg^−1^. Similarly, high ^210^Pb activities were also observed in cryoconite samples from a Swiss glacier, with a maximum of 4200 ± 240 Bq/kg as well as in other environmental matrices^13,58^. The presences of uranium and thorium are the main elements contributing to natural terrestrial radioactivity. Uranium isotopes (^234^U and ^238^U) in terrestrial samples (rocks, soils and sediments) are usually present in radioactive equilibrium. This equilibrium may be biased for samples from marine or freshwater environments. The main reason for radioactivity disequilibrium is greater mobility of ^234^U resulting in enriched ^234^U concentrations in waters and depleted ^234^U/^238^U activity ratios being observed for submerged solid samples. The main source of uranium in the natural environment is the atmospheric precipitation of terrigenic material, soil resuspension, rock weathering. The concentration of uranium can be increased by human activity (e.g. industry, fossil fuel combustion and industrial sewage)^59^. The value of the ^234^U/^238^U activity ratio in analysed cryoconite granules varies between 0.9 ± 0.1 to 1.1 ± 0.1, suggesting a state of radioactive equilibrium. Few studies have been performed on the equilibrium conditions of the ^232^Th decay chain in soils, but such equilibrium may be expected in most natural materials^60^. The activity concentrations of ^232^Th are also in agreement with cryoconite samples from Swiss glaciers^13^.

### Summary

Present study and previously published papers^11,13,40^ indicate that cryoconite holes are glacial reservoirs for heavy metals and radionuclides. Thus, new knowledge gaps have appeared; for example, the effect of glacial morphology on effective trapping and storing of radionuclides.

The differences in the concentrations of radionuclides between sampling points and the lack of clear differences in the elevation gradient from terminus towards icefall may reflect the heterogeneous topography of the glacier tongue. The surface of Adishi is wavy, with glacial wells, mills, and ablation forms^32^. There are many small grooves and gorges which may affect the accumulation of radioactivity on the surface.

The combination of the results obtained from different isotopic and mass ratios allowed us to investigate the proportion of Cs, Pu and Am from different sources. The main source of Pu is the global fallout, but the low ^240^Pu/^239^Pu atomic ratios seem to suggest the possibility of another, more local tropospheric source of ^239^Pu probably from the Kapustin Yar nuclear test site. High activity ratios of ^241^Am/^239+240^Pu could originate also from the local fallout from Kapustin Yar.

## Methods

### Sample collection and preparation

The structure and geological features of the Caucasian region of the Black Sea-Caspian Sea are determined by their location between the converging Eurasian and Africa-Arabian lithosphere plates within a zone of continent-continent collision^61^. The Caucasus Mountains located in this area are one of the main centers of mountain glaciation in Europe. The Greater Caucasus mountain range is located along the territory of Georgia, and it is divided into three parts, Western, Central and Eastern Caucasus^33,34^. At present, there are 637 glaciers in Georgia, and contemporary glaciers are mainly concentrated in the Enguri, Rioni, Kodori and Tergi river basins^32,33^. Adishi is a valley glacier with south-western exposition surrounded by roughed mountains (Fig. 1B). The area of Adishi glacier was 10.5 km^2^ and tongue was terminated on 2330 m. asl, in 1960. Adishi Glacier covers the area of 9.5 km^2^ and the terminus at 2,485 m asl^62,63^. The Glacier is divided into three parts: with the firn valley above 3800 meters, which is surrounded by the high peaks, grandiose icefall (~1000–1300 meters in height) and the classic ice tongue with a terminus at 2,485 m asl. The shape of the glacier changes dramatically from the ice base (2650 m), and the tongue is slightly inclined (~10°–15°)^64,65^. The surface of glaciers is wavy, with numerous glacial wells, mills and ablation forms. There are many small grooves and gorges in its surface formed by the melting water. Observation of the aerial images shows that the amount of weathered material has increased since 1960^32^.

Eight samples were collected in August 2014 from the Adishi Glacier (43°00′ N, 42°59′ E, c.a. 2 606 asl) from the ice tongue in the ablation zone. Samples 5 and 6 were collected from the same cryoconite hole. Samples from cryoconit reservoirs (Fig. 1C,D) with cryoconite granules (Fig. 1E) were collected to glass and plastic tubes, fixed with alcohol and transported to the laboratory. Then, samples were dried at 105 °C overnight and analysed using gamma (^137^Cs, ^210^Pb), alpha (^238^Pu, ^239+240^Pu, ^241^Am, ^234,238^U, ^230,232^Th), beta (^90^Sr) and mass (^240^Pu/^239^Pu) spectrometry.

### Analytical procedures for all isotopes

^137^Cs and ^210^Pb activities were determined using a planar HPGe (high-purity germanium) detector (home-made by the Institute of Nuclear Physics PAS Krakow and electronics by Silena S.p.A.). The activities of ^137^Cs were determined using its emission peak at 662 keV, and its emission peak at 46.6 keV was used to determine the activities of ^210^Pb. The absolute efficiencies of the detector were determined using calibrated sources and sediment samples of known activity. Also, corrections were made to measure the effect of self-absorption of low-energy γ-rays (46.6 keV) within the sample, although these corrections were insignificant because the masses of the samples were low. The activities of the ^238^Pu, ^239+240^Pu, ^241^Am, ^234,238^U, ^230,232^Th and ^90^Sr dried samples were determined 0.94 and 1.93 g. Organic matter in the samples was decomposed by heating in a Muffle oven at 600 °C for 6 hours.

The samples were dissolved using concentrated HF, HNO_3_, HCl, and a small addition of H_3_BO_3_. Details of the sequential radiochemical procedure used to determine ^238^Pu, ^239+240^Pu, ^241^Am, ^234,238^U, ^230,232^Th and ^90^Sr are described in previous publications^64,65^. Also, the measurements of plutonium and americium isotopes activities were determined using alpha particle spectrometers with passivated planar silicon (PIPS) detectors (Canberra) on a Silena Alphaquattro spectrometer (Silena S.p.A). ^90^Sr was measured using a Wallac 1414 Guardian LSC spectrometer for the equilibrated ^90^Sr–^90^Y fraction after determining the chemical recovery of ^85^Sr by gamma-spectrometry. The full sequential radiochemical procedure and gamma analyses were verified using soil reference material produced by the International Atomic Energy Agency (IAEA 447). After the alpha-spectrometric measurements, the Nd(Pu)F_3_ alpha-spectrometric sources in the form of polyvinyl chloride filters glued to stainless steel planchettes were removed by immersing them in a small volume of warm water. After the filters were separated from the supporting metal disks, the disks were removed and the water was evaporated to dryness. To the dried filters, 0.5 g of solid H_3_BO_3_ and the portions of Aqua Regia (5 ml each) were added and evaporated to dryness. The remainder of the filters was ignited at 450 °C to remove the organic material, and the residue was further attacked with approx. 0.5 ml of concentrated HClO_4_. After evaporation to dryness, the samples were dissolved in 5 ml of concentrated HCl, 10 mg of Fe carrier was added and the samples were transferred to centrifuge tubes followed by the MQ water wash to ensure the quantitative transfer. Pu was pre-concentrated by Fe(OH)_3_ precipitation using ammonia solution. The precipitate was then separated from the liquid by centrifuging and the supernatant was discarded. The residual precipitate was dissolved in 9 M HCl and loaded onto an anion exchange resin (Eichrom 1 × 8 Cl form, 100–200 mesh). Pu was retained on the column and washed with 30 ml of 9 M HCl followed by 2 × 15 ml of 8 M HNO_3_. Finally, an additional 10 ml of 9 M HCl was loaded to the column to converted back to Cl form, and Pu fraction was eluted using 30 ml of freshly prepared 9 M HCl/NH_4_I solution. The purified Pu fractions were evaporated to dryness by adding 5 ml of concentrated HNO_3_ to remove the excess iodide presence and transferring them into analytical vials using 1 ml of 2% (v/v) nitric acid. The samples were later analysed using Neptune MC-ICP-MS and calibrated with standards of known ^239^Pu and ^240^Pu concentrations.

The reference data for ^210^Pb, ^137^Cs and ^90^Sr activity August 2014 year.

The percentage of the global fallout of Pu isotopes versus Chernobyl fallout was estimated using the following formula described by Mietelski and Wąs^66^:

1 $${f}_{G}=({A}_{R}-{A}_{M})/({A}_{R}-{A}_{G})$$

where f_G_ represents the fraction of plutonium isotopes from global fallout sources (%), A_R_ represents the ^238^Pu/^239+240^Pu ratio of Chernobyl sources (0.45), A_M_ represents the measured ^238^Pu/^239+240^Pu activity ratio in cryoconite samples and A_G_ represents the ^238^Pu/^239+240^Pu activity ratio of global fallout (0.025).

## Electronic supplementary material

Table S1, Table S2, Table S3
