# Supplementary material for: The sources of high airborne radioactivity in cryoconite holes from the Caucasus (Georgia)
Source: Sci Rep. 2018 Jul 17;8:10802. doi: 10.1038/s41598-018-29076-4 (PMC6050279; doi:10.1038/s41598-018-29076-4)
Supplement: Supplementary file 1 — Table S1, Table S2, Table S3 [file 41598_2018_29076_MOESM1_ESM.docx]

**The sources of high airborne radioactivity in cryoconite holes from southern Caucasus (Georgia)**

**Edyta Łokas^1^**^*^**, Krzysztof Zawierucha^2^, Anna Cwanek^1^, Katarzyna Szufa^1^, Paweł Gaca^3^, Jerzy W. Mietelski^1^, Ewa Tomankiewicz^1^**

1 Department of Nuclear Physical Chemistry, Institute of Nuclear Physics Polish Academy of Sciences, Kraków, Radzikowskiego 152, 31-342, Poland

2 Department of Animal Taxonomy and Ecology, Adam Mickiewicz University in Poznań, Poland

3 GAU-Radioanalytical Laboratories, Ocean and Earth Science, University of Southampton, National Oceanography Centre, European Way, Southampton, United Kingdom

* corresponding author, email: [*Edyta.Lokas@ifj.edu.pl](mailto:*Edyta.Lokas@ifj.edu.pl)

**Tab. S1. Activity concentrations of anthropogenic radionuclides (^137^Cs, Pu isotopes, ^241^Am, ^90^Sr) expressed in Bq kg^-1^ and organic matter in all cryoconite samples. All data were corrected for August 2014, the sampling date.**

| No. | ^137^Cs (Bq/kg) | ^239+240^Pu (Bq/kg) | ^238^Pu (Bq/kg) | ^241^Am  (Bq/kg) | ^90^Sr  (Bq/kg) | Organic matter (%) |
| --- | --- | --- | --- | --- | --- | --- |
| 1 | 2250 ± 290 | 52.9 ± 3.6 | 1.48 ± 0.16 | 42.9± 2.8 | 97 ± 8 | 11 |
| 2 | 4900 ± 600 | 107.3 ± 6.9 | 2.52 ± 0.20 | 68.3 ± 4.3 | 68 ± 5 | 16 |
| 3 | 1080 ± 140 | 11.6 ± 0.8 | 0.37 ± 0.06 | 12.0 ± 0.7 | 37 ± 3 | 4 |
| 4 | 580 ± 80 | 10.2 ± 0.8 | 0.24 ± 0.05 | 8.7 ± 0.5 | 23 ± 2 | 3 |
| 5 | 2680 ± 330 | 33.6 ± 2.2 | 1.06 ± 0.10 | 32.7 ± 2.0 | 37 ± 3 | 9 |
| 6 | 3000 ± 380 | 36.3 ± 2.5 | 1.03 ± 0.11 | 33.6 ± 2.1 | 54 ± 4 | 10 |
| 7 | 3360 ± 420 | 18.8 ± 1.3 | 0.81 ± 0.09 | 16.2 ± 0.8 | 53 ± 4 | 10 |
| 8 | 3570 ± 450 | 10.0 ± 0.7 | 0.37 ± 0.04 | 8.1 ± 0.4 | 31 ± 2 | 10 |

**Tab. S2. Radionuclide activity ratios and mass ratios for all cryoconite samples.**

| No. | ^238^Pu/^239±240^Pu | ^239±240^Pu/^137^Cs | ^241^Am/^239±240^Pu | ^240^Pu/^239^Pu | ^90^Sr/^137^Cs | ^90^Sr/^239+240^Pu |
| --- | --- | --- | --- | --- | --- | --- |
| 1 | 0.028 ± 0.004 | 0.024 ± 0.004 | 0.89 ± 0.08 | 0.161 ± 0.002 | 0.043 ± 0.007 | 1.83± 0.12 |
| 2 | 0.023 ± 0.002 | 0.022 ± 0.003 | 0.61 ± 0.05 | 0.132 ± 0.001 | 0.014 ± 0.002 | 0.63 ± 0.14 |
| 3 | 0.032 ± 0.005 | 0.011 ± 0.002 | 0.95 ± 0.09 | 0.169 ± 0.001 | 0.035 ± 0.005 | 3.22 ± 0.23 |
| 4 | 0.024 ± 0.005 | 0.018 ± 0.003 | 0.88 ± 0.08 | 0.177 ± 0.003 | 0.040 ± 0.007 | 2.28 ± 0.17 |
| 5 | 0.032 ± 0.004 | 0.013 ± 0.002 | 0.94 ± 0.08 | 0.169 ± 0.001 | 0.014 ± 0.002 | 1.11 ± 0.07 |
| 6 | 0.028 ± 0.004 | 0.012 ± 0.002 | 0.99 ± 0.09 | 0.168 ± 0.001 | 0.018 ± 0.003 | 1.47 ± 0.10 |
| 7 | 0.043 ± 0.005 | 0.006 ± 0.001 | 0.76 ± 0.05 | 0.154 ± 0.002 | 0.016 ± 0.002 | 2.84 ± 0.19 |
| 8 | 0.037 ± 0.005 | 0.003 ± 0.001 | 0.88 ± 0.08 | 0.172 ± 0.004 | 0.009 ± 0.001 | 3.11 ± 0.20 |

| No. | ^210^Pb  (Bq/kg) | ^238^U  (Bq/kg) | ^234^U  (Bq/kg) | ^230^Th  (Bq/kg) | ^232^Th  (Bq/kg) | ^234^U/^238^U |
| --- | --- | --- | --- | --- | --- | --- |
| 1 | 2600 ± 250 | 33 ± 3 | 38 ± 3 | 64 ± 5 | 54 ± 5 | 1.1 ± 0.1 |
| 2 | 4100 ± 200 | 32 ± 4 | 34 ± 3 | 49 ± 4 | 44 ± 4 | 1.0 ± 0.2 |
| 3 | 2100 ± 150 | 44 ± 4 | 39 ± 3 | 54 ± 4 | 41 ± 3 | 0.9 ± 0.1 |
| 4 | 1400 ± 100 | 35 ± 3 | 32 ± 3 | 50 ± 4 | 35 ± 3 | 0.9 ± 0.1 |
| 5 | 4600 ± 200 | 29 ± 3 | 27 ± 2 | 42 ± 3 | 37 ± 3 | 0.9 ± 0.1 |
| 6 | 5500 ± 300 | 30 ± 3 | 30 ± 2 | 44 ± 3 | 38 ± 3 | 1.0 ± 0.1 |
| 7 | 9300 ± 500 | 35 ± 4 | 33 ± 3 | 53 ± 4 | 45 ± 4 | 0.9 ± 0.1 |
| 8 | 12000 ± 600 | 33 ± 3 | 33 ± 3 | 49 ± 4 | 41 ± 3 | 1.0 ± 0.1 |

**Tab. S3. Activity concentrations of natural radionuclides (^210^Pb, ^234,238^U, ^230,232^Th) expressed in Bq kg^-1^ and activity ratio of ^234^U/^238^U in all cryoconite samples. Data for ^210^Pb were corrected for August 2014, the sampling date.**
